# Embedding of Ultrathin Chips in Highly Flexible, Photosensitive Solder Mask Resist

**DOI:** 10.3390/mi12080856

**Published:** 2021-07-21

**Authors:** Florian Janek, Nadine Eichhorn, Sascha Weser, Kerstin Gläser, Wolfgang Eberhardt, André Zimmermann

**Affiliations:** 1 Hahn-Schickard, Allmandring 9B, 70569 Stuttgart, Germany; Nadine.Eichhorn@hahn-schickard.de (N.E.); Sascha.Weser@hahn-schickard.de (S.W.); Kerstin.Glaeser@hahn-schickard.de (K.G.); Wolfgang.Eberhardt@hahn-schickard.de (W.E.); Zimmermann@ifm.uni-stuttgart.de (A.Z.); 2 Institute for Micro Integration (IFM), University of Stuttgart, Allmandring 9B, 70569 Stuttgart, Germany

**Keywords:** system-in-foil, ultrathin chips, embedding technology, inkjet printing, flexible system

## Abstract

This work presents an embedding process for ultrathin silicon chips in mechanically flexible solder mask resist and their electrical contacting by inkjet printing. Photosensitive solder mask resist is applied by conformal spray coating onto epoxy bonded ultrathin chips with a daisy chain layout. The contact pads are opened by photolithography using UV direct light exposure. Circular and rectangular openings of 90 µm and 130 µm diameter, respectively, edge length are realized. Commercial inks containing nanoparticular silver and gold are inkjet printed to form conductive tracks between daisy chain structures. Different numbers of ink layers are applied. The track resistances are characterized by needle probing. Silver ink shows low resistances only for multiple layers and 90 µm openings, while gold ink exhibits low resistances in the single-digit Ω-range for minimum two printed layers.

## 1. Introduction

The demand for high functional density of microelectronics is accompanied by constant downsizing of components and their sophisticated packaging technologies. This led to thinning of silicon chips for a further size reduction down to 50 µm thickness and less, achieving ultrathin chips. At these low thicknesses, the silicon becomes mechanically flexible [1,2]. This characteristic can be used to integrate ultrathin silicon microchips into polymer layers resulting in a mechanically bendable system-in-foil (SiF) with a very low total thickness and low mass. This makes it possible to utilize SiF in narrow installation spaces for sensorization, for medical applications such as implants or on skin, as well as smart wearables in combination with textiles.

Various publications show production methods for SiF. The methods can be categorized by the placement orientation of the chip on the substrate. One approach is to utilize a flip-chip process [3,4,5,6,7,8,9]. The chip is placed with its contact site towards prepared circuit tracks, also called the face-down approach. The mechanical fixation and the electrical connection are performed simultaneously using a conductive adhesive. An encapsulation layer is applied for protection, planarization and reduction in mechanical stress on interconnections and the chip. This process requires an additional process step to apply electrically conductive bumps on the contact pads of the silicon chip to establish an electrical path onto the premade tracks on the surface of the substrate. The bumps complicate the delicate handling process of the ultrathin chips further, and can lead to mechanical stress onto the brittle silicon and risk fracturing of the chips [5,10]. 

The other approach is called face-up placement. The bonding of the chips and the metallization step are performed successively. In the first instance, the backside of an ultrathin chip is adhesively bonded onto a substrate. After bonding, the contact pads are available for electrical connection. Either the chip contact pads are contacted directly by forming conductive tracks on the chip’s surface or a polymer layer is applied. The choice of adhesives for the bonding process depends on the substrate material but also on the used polymer layer and its curing temperature. Often, polyimide is used as a polymer layer due to its superior thermal, chemical and mechanical stability and its excellent mechanical flexibility after curing. On the other side, the curing of polyimide requires an oxygen-free atmosphere and high temperature up to 350 °C for complete curing [11]. Often, benzocyclobutene (BCB) is utilized as an adhesive due to its high thermal stability and strong adhesion to silicon [12]. Polyimide, as well as BCB, can be applied by spin coating, resulting in thin layers with homogenous thicknesses [13,14,15]. Openings in the polymeric layer can be established by laser drilling [10,16,17,18,19] or photolithography [20,21,22]. Afterwards, conductive tracks are applied. Possible processes for track formation are printing of inks filled with metal nanoparticles [23,24] or physical vapor deposition (PVD) of metal layers [13,25,26,27] in combination with photolithography processes for structuring. 

Despite of the apparent advantages of SiF for a broad range of applications and various known methods for the embedding of ultrathin chips and their electrical contacting, the breakthrough for products utilizing ultrathin chips has not happened yet. The most likely reasons are the high manufacturing costs of SiF, especially in the case of prototypes and its small quantities. This publication presents results from ongoing research on a novel approach for the coating of face-up bonded chips. This process utilizes conformal spray coating of a mechanically flexible solder mask resist for embedding adhesively fixed chips on foil substrates. Solder mask resist is cheaper than polyimide, has excellent adhesion to silicon and the curing can be performed at 150 °C, which is significantly lower than the required 350 °C of polyimide. Furthermore, the curing of solder mask resist can be performed in air atmosphere, in contrast to the oxygen-free atmosphere required for the imidization process of polyimide. The solder mask resist is structured by a UV direct light photolithography process to open via to the chip contact pads. Electrically conductive tracks are printed directly onto the substrate by inkjet printing of nanoparticular silver ink or nanoparticular gold ink to electrically connect the opened contact pads. The transition resistances are measured and the achieved values are compared for both inks as well as for different opening dimensions, opening shapes and printed layer numbers.

## 2. Materials and Methods

**Figure 1:**
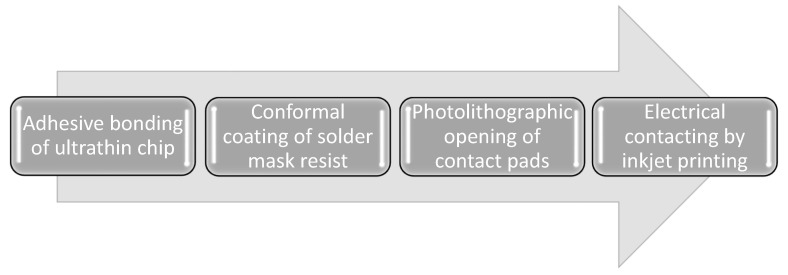
Process flow chart of the proposed process for the fabrication of SiF.

A process flow chart of the proposed fabrication process is shown in Figure 1. In the beginning, ultrathin chips were bonded face-up onto polyimide foil. Afterwards, the chip was covered in solder mask resist using conformal coating. Solder mask resist was locally removed in the area of the chip contact pads by photolithographic processes and cured. The chip contact pads were electrically connected and conductive tracks were formed by inkjet printing of silver and gold ink. Each element of the proposed process is explained in detail in the following subsection.

### 2.1. Silicon Chip Design and Substrate Preparation

Silicon chips of 4.7 mm × 4.7 mm size were manufactured, thinned to 50 µm thickness and supplied ready for the pick and place processes by IMS Chips, Stuttgart, Germany. The chip layout exhibits 210 resistors, which can be connected in a “daisy chain” series circuit. The ultrathin chips were transferred face-down on a wafer dicing tape (REVALPHA Tape 3196, Nitto Denko, Osaka, Japan). An epoxy adhesive (EPO-TEK 354, Epoxy Technology, Billerica, MA, USA) was manually dispensed onto the backside of the chip. A total of 75 µm polyimide foil (Flexiso PI FI 16000, Dr. Dietrich Müller GmbH, Ahlhorn, Germany), temporarily fixed on a stiff handling substrate, was placed onto the stack of wafer dicing tape, ultrathin chip and epoxy adhesive. The epoxy adhesive was cured in an oven at 80 °C for 120 min. The adhesion force of the wafer dicing tape to the silicon chip was strongly reduced when exposed to 80 °C for the time of adhesive curing and the wafer dicing tape was peeled off by hand. The surface of the ultrathin chips was cleaned with acetone and cleanroom tissue. 

### 2.2. Conformal Coating of Solder Resist

The negative-tone solder mask resist (NPR-80/ID100, Nippon Polytech Corp, Tokyo, Japan) used in this work was manufactured for screen printing as a two component system with a mixing ratio of 100 g resin to 38 g corresponding hardener PF-10/ID36. Both components were weighted according to the recommended mixing ratio and stirred for five minutes. To achieve a lower viscosity suitable for conformal coating, the mixture was diluted 1:1.25 wt% with the solvent propylene glycol methyl ether acetate (PGMEA). The dilution was mixed for five minutes and poured into a 30 mL cartridge sealed with a plunger. The cartridge, together with a barrel adapter, was installed in a conformal coating machine (ELITE DR-070, Nordson DIMA BV, Deurne, The Netherlands). A narrow beam spray coating valve (Type DD-5140, Nordson DIMA BV, Deurne, The Netherlands) was used to achieve a layer thickness of 15–20 µm as recommended in the technical data sheet of the solder mask resist. Empirical trials showed that a dilution ratio of 1:1.25 wt% (NPR-80/ID100: PGMEA) yielded the best tradeoff between application pressure and mixture stability. Lower amounts of PGMEA obtained the high application pressure necessary with the difficulty to obtain the targeted thickness range. Higher amounts of PGMEA led to faster separation of solder mask resist and PGMEA inside the cartridge. In the case of separation, the cartridge can be removed, shaken for five minutes, and re-installed. 

During the application process, the valve was moved in multiple parallel tracks in a distance of 20 mm above the substrate with a distance of 15 mm between each track. The application pressure on the plunger was adjusted to 1.5 bar. The atomizer pressure, necessary for the generation of an aerosol for the spray coating process, was set to 0.5 bar. The movement velocity of the valve was set to 450 mm/s. The substrates were laid onto a substrate table heated to 50 °C. The coated substrates stayed for five minutes on the heated table for evaporation of solvent. The substrate was transferred onto an 80 °C hotplate for additional ten minutes for the evaporation of the remaining solvent. Table 1 summarizes the parameters for the conformal spray coating process to achieve a layer thickness of ca. 15 µm. 

### 2.3. Exposure and Development

**Figure 2:**
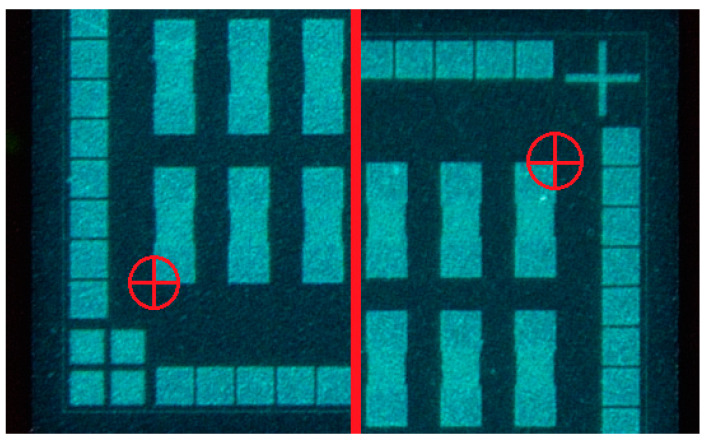
Representation of virtual marker in opposite chip corners for layout alignment during direct light imaging UV exposure of solder mask resist.

The coated substrates with embedded ultrathin chips were exposed to UV light using a direct light imager system (DI 2020L, MIVA Technologies, Schönaich, Germany) to open the metallic surface of the chip contact pads. The imaging system did not require a physical mask for the UV exposure process. A machine head with built-in UV-LEDs was moved above the vacuum-fixed substrate. Four different wavelengths, namely 365 nm, 375 nm, 385 nm and 405 nm, could be used simultaneously as well as individually for the exposure process. For the exposure of the solder mask resist, all wavelengths were used in equal dose proportions. The exposure dose was adjusted by movement velocity of the head as well as the LED current for each wavelength. For the exposure of the coated substrates, the head movement velocity and the LED currents were set to parameters that equaled an exposure dose of 1130 mJ/cm^2^. Two layouts for the exposure were designed with different via sizes and geometries using conventional PCB layout software. Each layout was divided into circular contact pad openings in the chips upper half and rectangular contact pad openings in the chips lower half. One layout generated circular via openings of 90 µm diameter and rectangular via openings of 90 µm × 90 µm. The other layout generates circular via openings of 130 µm diameter and rectangular via openings of 130 µm × 130 µm. The size of 90 µm was chosen to stay above the stated technical resolvable limit of 80 µm for photolithographic opening size as stated by the manufacturer of the solder mask resist [28]. The size of 130 µm was chosen arbitrarily to test ink behavior during and after printing with larger via openings. Areas for exposure as well as virtual markers in two opposite chip corners (Figure 2) are defined within the layouts. The markers served as fiducial points for optical recognition before each UV exposure to ensure alignment. The exposed negative-tone resist was allowed to cross-link for five minutes after UV exposure. The development process was executed for a duration of 30 s in a spray developer using aqueous potassium carbonate solution at 30 °C. The substrate was cleaned afterwards using deionized water. The photolithographic process was completed by thermal curing in an oven at 150 °C for 60 min. The summarized exposure, development and thermal curing parameters are listed in Table 2. 

### 2.4. Application of Conductive Tracks by Inkjet Printing

**Figure 3:**
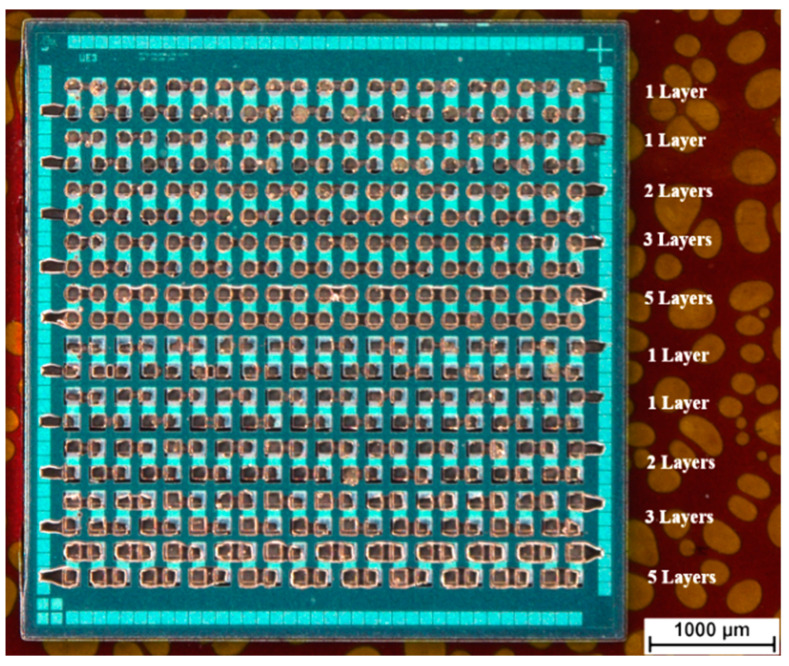
Overview of printed layers for each daisy chain line. First and sixth line were printed for alignment with one layer each. The other lines were printed for electrical measurement with varying number of layers.

Inkjet printing of commercial nanoparticular inks was utilized for electrical contacting of the chip contact pads. A silver ink (Sicrys I30EG-1, PV Nano Cell, Migdal Ha’Emek, Israel) as well as a gold ink (DryCure Au-J, C-INK, Soja City, Japan) were printed using a lab inkjet printer (Dimatix DMP 2850, FUJIFILM Dimatix, Santa Clara, CA, USA) for via filling and track formation. An amount of 2 mL of ink was filled into cartridges (DMC 11610, Fujifilm, Japan). A filter with 450 nm pore size was used for the silver ink to filter out agglomerates. The gold ink was filled into the cartridge without filtering. A 10 pL print head module with 16 nozzles was mounted onto each cartridge and the cartridges were installed into the printer. A layout for printing was designed using a conventional PCB layout software. The layout was exported in a monochrome bitmap file with 1016 dpi resolution. This resolution equals a drop spacing of 25 µm at a cartridge angle of 5.6°. The surface of the substrate was cleaned with acetone and a cleanroom tissue. For the printing process of both inks, the print head was heated to 35 °C and the temperature of the substrate table was set to 50 °C. Only one nozzle of 16 potential nozzles was used to perform the print to allow for fast visible control if the printing nozzle clogs or the ejected droplets are not shot perpendicular to the surface. The same layout for track printing and via filling was used for the rectangular and the circular via openings. The number of layers was varied between the daisy chain structure lines. In total, ten daisy chain lines were available for printing. The first daisy chain line and the sixth daisy chain line were printed as a single ink layer to check for correct alignment. For later electrical measurements the other daisy chain lines were printed as a single ink layer, two ink layers, three ink layers and five ink layers. This was set as printing strategy for the circular as well as for the rectangular via openings. An overview over the printed layer count is displayed in Figure 3. After the printing process, the solvent was mostly evaporated due to the heated substrate table. The printed nanoparticles were sintered in an oven for 60 min at 150°. The electrical characterization was performed by needle probing in a two-probe electrical measurement (Multimeter 2002, Keithley Instruments, Cleveland, OH, USA) to determine the transition resistance. Additionally, white light interferometry (Wyko NT 9100, Bruker, Billerica, MA, USA) was performed to determine the thickness distribution of printed tracks. Finally, the foil with the embedded chip was removed from the temporary carrier.

## 3. Results

**Figure 4:**
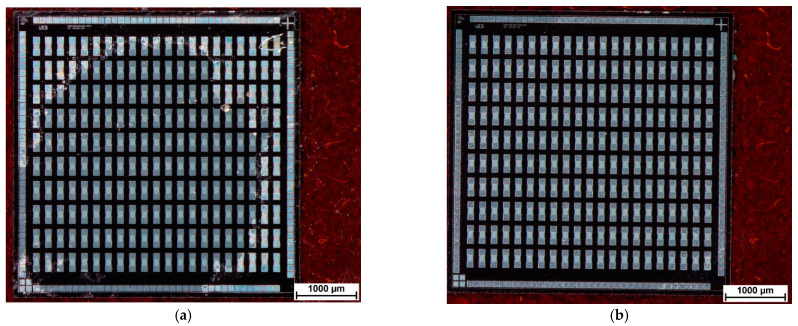
(**a**) Ultrathin chip epoxy bonded on polyimide foil. Remaining adhesive from wafer dicing tape is visible on the surface after removing the wafer dicing tape. (**b**) Surface of epoxy bonded ultrathin chip after acetone cleaning.

**Figure 5:**
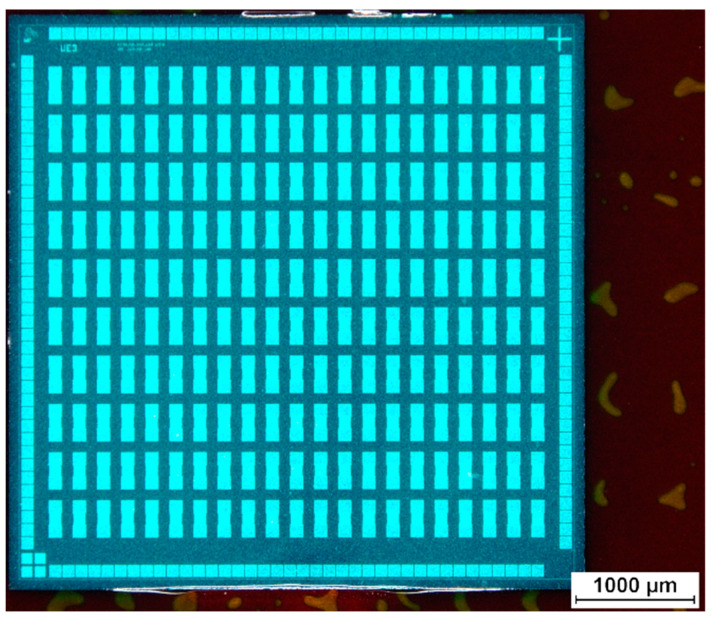
Ultrathin chip with daisy chain structures embedded in solder mask resist. The bubbles visible in the background right from the chip are located in an adhesive layer. This adhesive layer is used as temporary fixation of a polyimide foil on a stiff handling substrate.

The wafer dicing tape can be removed easily without damages to the silicon chips. Remaining adhesive from the wafer dicing tape on the surface of the epoxy bonded chips was mechanically cleaned using acetone and clean room tissues. A chip on polyimide foil after acetone cleaning is displayed in Figure 4. Further details about the ultrathin chip bonding process using a wafer dicing tape as a temporary chip carrier can be found in [29,30]. After cleaning, a layer of solder mask resist was spray coated onto the ultrathin chip. Figure 5 shows the surface of an ultrathin chip embedded in spray coated solder mask resist. 

**Figure 6:**
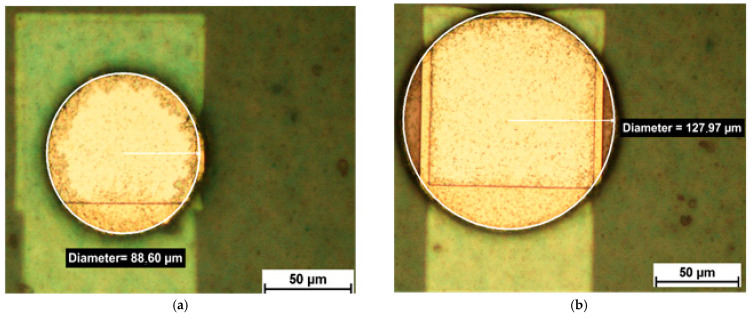
Circular via openings with (**a**) a diameter of 90 µm and (**b**) a diameter of 130 µm.

**Figure 7:**
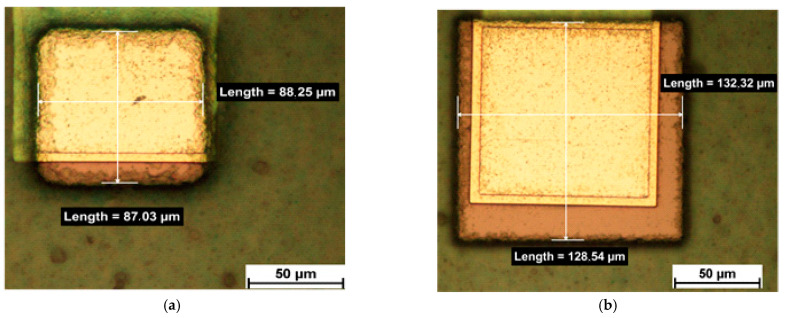
Rectangular via openings of (**a**) 90 µm × 90 µm and (**b**) 130 µm × 130 µm.

**Figure 8:**
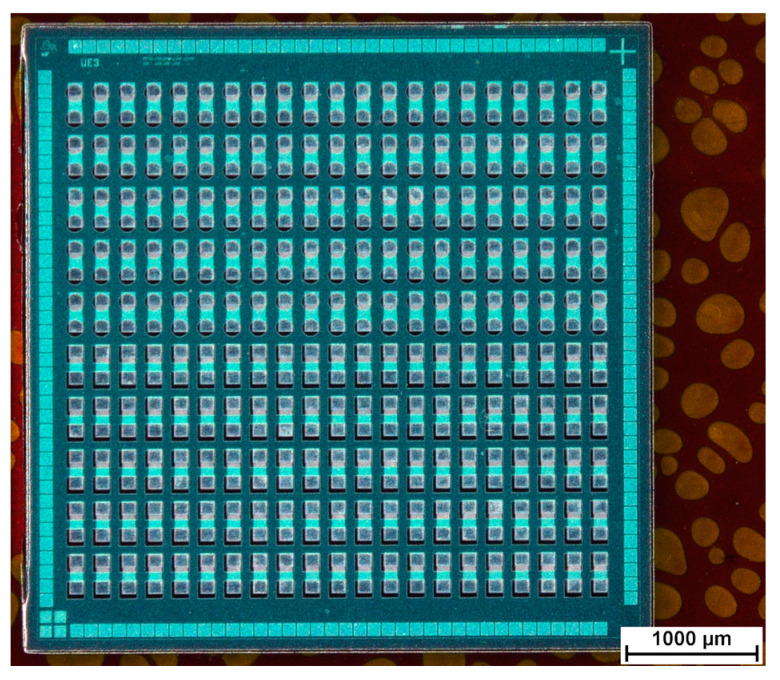
Overall view on an ultrathin chip embedded in solder mask after photolithographic structuring. The opening dimensions are 130 µm. The bubbles visible in the background are located in an adhesive layer. This adhesive layer is used as temporary fixation of a polyimide foil on a stiff handling substrate.

The different via openings after photolithography are shown in Figure 6 and Figure 7. Figure 6 shows circular via openings of 90 µm diameter and 130 µm diameter, respectively. Figure 7 shows rectangular via openings of 90 µm × 90 µm and 130 µm × 130 µm, respectively. Figure 8 shows an overview of an embedded chip with circular via openings in the upper half and rectangular via openings in the lower half with 130 µm feature sizes.

**Figure 9:**
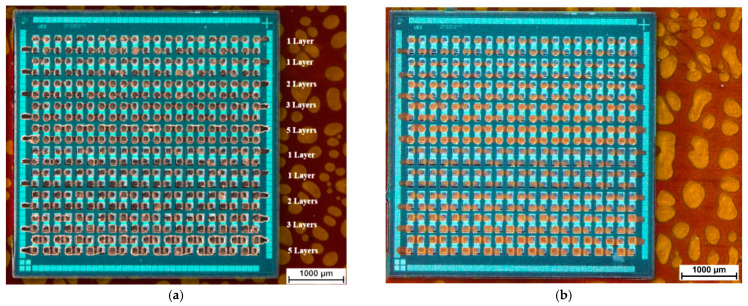
Overall view after printing and sintering of (**a**) silver ink and (**b**) gold ink. The notation displays the numbers of printed layers for each daisy chain line.

**Figure 10:**
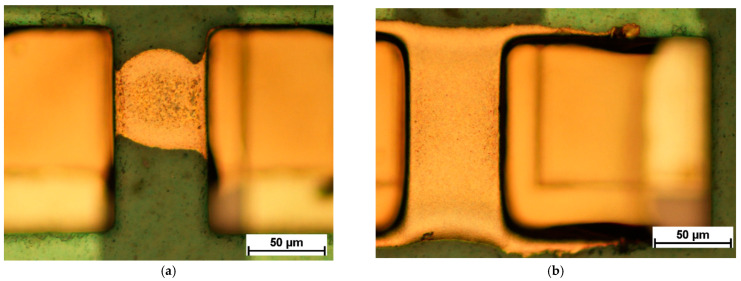
Connection between rectangular via openings of 130 µm × 130 µm size. (**a**) One layer of printed silver ink after sintering. (**b**) Five layers of printed silver ink after sintering.

**Figure 11:**
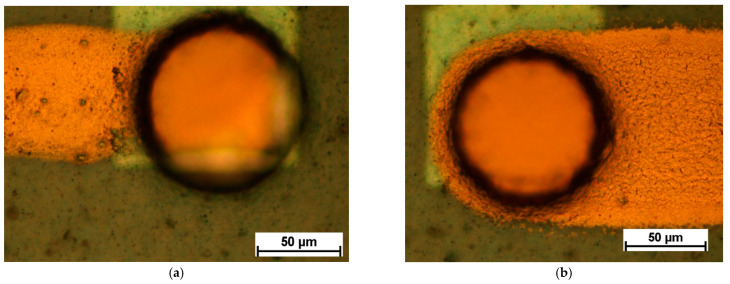
Connection between circular via openings of 90 µm diameter. (**a**) One layer of printed gold ink after sintering. (**b**) Five layers of printed gold ink after sintering.

Figure 9 shows an overview over embedded ultrathin chips with 130 µm via opening sizes after silver ink and gold ink inkjet printing. Figure 10 demonstrates the difference between one printed silver layer and five printed silver layers for 130 µm × 130 µm rectangular via openings after sintering the silver ink. Figure 11 demonstrates the difference between one printed gold layer and five printed gold layers for 90 µm circular via openings after sintering the gold ink.

**Figure 12:**
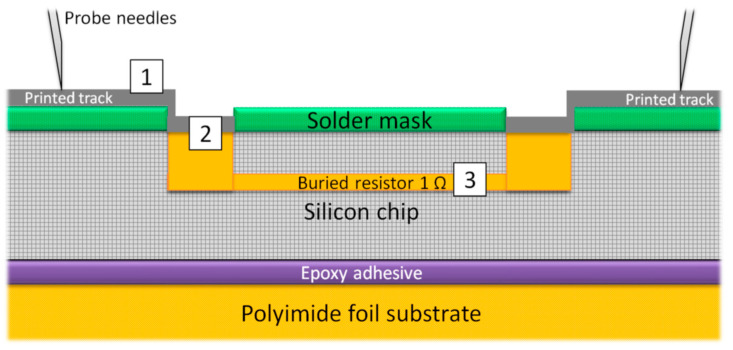
Schematic layer structure (not to scale) of an epoxy bonded chip on polyimide substrate and embedded in solder mask. The chip exhibits buried resistors (1 Ω). The contacts pads of the buried resistors were opened and contacted by inkjet printing. Mainly three factors influence the transition resistance from the first probe needle to the other probe needle. (1) Resistance of the printed track; (2) contact resistance at the interface; (3) the resistance of the buried resistor.

The layer structure, presented in Figure 12, shows the three factors that influence the transition resistance from one probe needle to the other probe needle. The first factor is the resistance of the ink itself. The second factor is the contact resistance at the interface ink to contact pad. The third factor is the resistance of the buried resistor. The nominal value of resistance of the buried resistor is 1 Ω. The mean resistance values and standard deviation of the measured transition resistances for rectangular and circular via openings with varying sizes and different layer count are summarized in Table 3 for the silver ink and in Table 4 for the gold ink. Each entry shows the mean resistance value and standard deviation for a daisy chain line with 21 transitions. It is noted if a transition was not measurable due to a defect by an interruption in the printed track. In this case, the mean values and standard deviation values were calculated from the remaining transition structures. 

**Figure 13:**
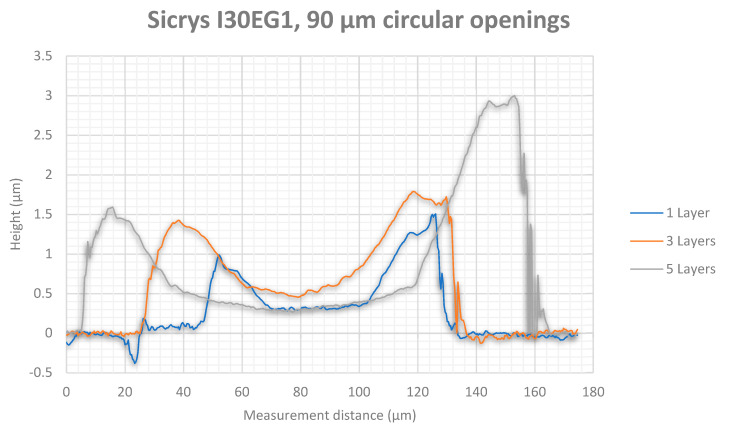
White light interferometry measurement of the silver track thickness between circular via openings with 90 µm diameter after 1, 3 and 5 printed layers.

**Figure 14:**
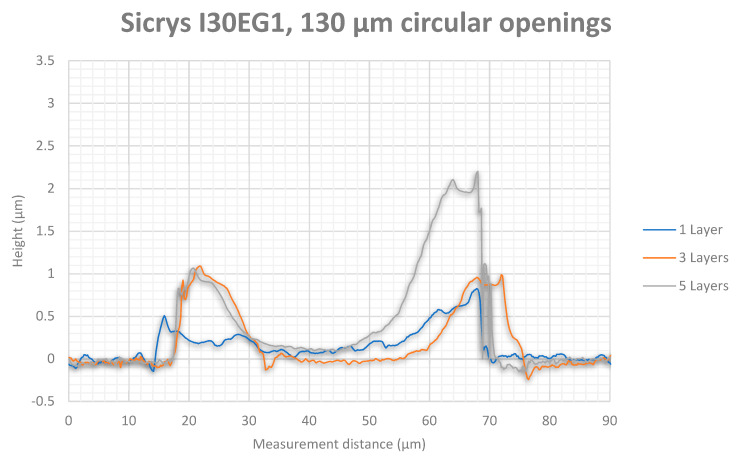
White light interferometry measurement of the silver track thickness between circular via openings with 130 µm diameter after 1, 3 and 5 printed layers.

**Figure 15:**
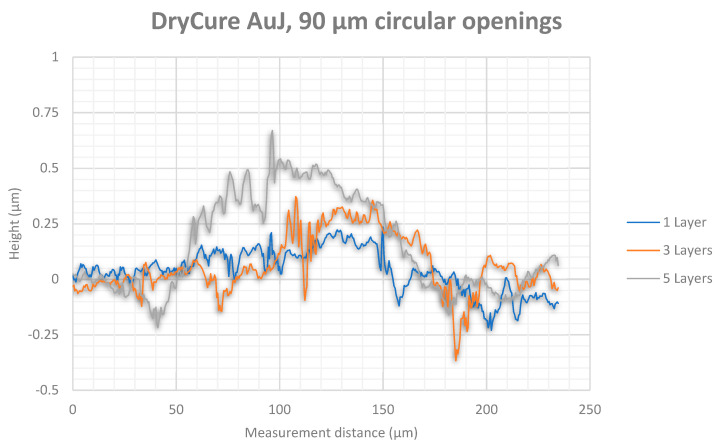
White light interferometry measurement of the gold track thickness between circular via openings with 90 µm diameter after 1, 3 and 5 printed layers.

**Figure 16:**
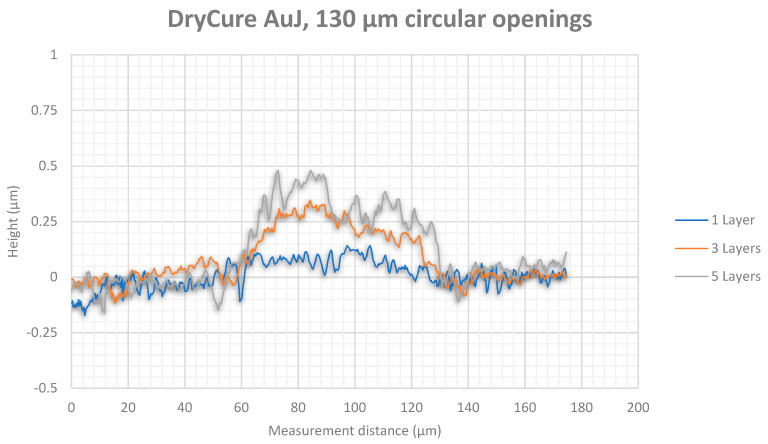
White light interferometry measurement of the gold track thickness between circular via openings with 130 µm diameter after 1, 3 and 5 printed layers.

White light interferometry measurement results showed higher thicknesses for the silver tracks (Figure 13 for 90 µm diameter and Figure 14 for 130 µm diameter) than for the gold tracks (Figure 15 for 90 µm diameter and Figure 16 for 130 µm diameter). Furthermore, silver nanoparticles rather gather in the peripheral region in contrast to gold nanoparticles that concentrate in the center of the track.

**Figure 17:**
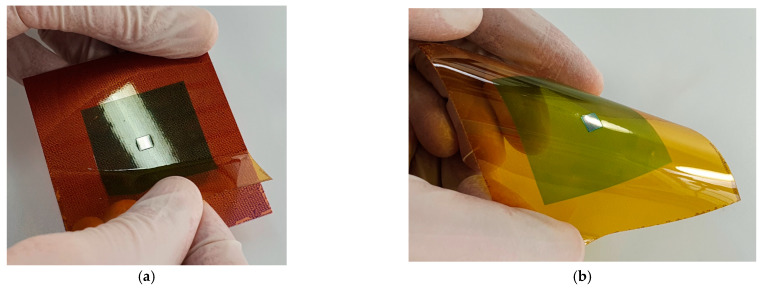
(**a**) Removing of polyimide foil substrate by peeling off of the temporary carrier substrate. (**b**) Mechanically flexible ultrathin chip embedded in solder mask resist.

The removal of the foil with the embedded chip from the temporary carrier was performed by peeling (Figure 17). The solder mask as well as the ultrathin chip are mechanically flexible.

## 4. Discussion

The manufacturer of the solder mask resist intended the application by screen printing. To avoid the need for a screen, the solder mask resist application was transferred to a conformal coating machine. For a more versatile spray coating process, the solder mask resist mixture was diluted using PGMEA, a standard solvent in photolithography technology. A combination of a mixing ratio, application pressure and valve movement velocity generated a homogeneous layer without defects. The thickness of the solder mask layer can be influenced by the adjustment of the valve movement velocity. Conformal coating allows for a locally defined application of resist saving material and reducing cleaning expenses compared to the full area coverage of spin coating. Further arbitrary substrate geometries can be coated, while spin coating is limited to round substrates.

For the UV exposure of the photosensitive solder mask resist, a dose of 1130 mJ/cm^2^ was found to be sufficient to start the cross-linking reactions in the material. The most influential factor on the resulting resist mask was the development duration. On the one hand, the duration of the development process must be chosen long enough to remove all insulating solder mask material on the chip contact pads. On the other hand, the edge formation of the solder mask was deteriorated if the sprayed developer reacted for too long with the solder mask. Instead of the recommended 60 s, the development duration was reduced to 30 s. All contact pad openings were successfully opened after development. This demonstrated that thinning with PGMEA is possible. In comparison with conventional mask photolithography, the utilization of direct light imaging offers a significantly faster development process of new prototypes. The layout can be altered digitally in a short time without an additional costly fabrication of a new set of masks. 

The white light interferometry measurements show a clearly noticeable coffee-stain effect for the silver ink, where the nanoparticles were affected by a forced flow towards the peripheral regions of the wetted area due to a gradient of surface tension. In contrast, the gold particles were concentrated in the center of the printed track due to a Marangoni flow. Furthermore, the higher content of silver nanoparticles (30 wt%) in the silver ink Sicrys I30EG-1 compared to gold nanoparticles (10 wt%) within the gold ink DryCure Au-J, contributed to a higher track thickness of printed silver. 

The measurements of transition resistances listed in Table 3 and Table 4 show mixed results. It should be noted that inhomogeneous track thicknesses affected the measured resistance values during needle probing. A placement of the needles in the peripheral region of the printed track showed different resistance values than a measurement in the center of the printed tracks dependent on the printed ink. For silver ink, a smaller opening dimensions showed lower resistance values and no defects in comparison to larger via openings with multiple defects. Gold ink did not show this distinct difference. This may result from a combination of different wetting behaviors of the inks and residual solder mask resist after development in the 90 µm via openings that served as a ramp for the inks. The silver ink Sicrys I30EG-1 was based on ethylene glycol as the solvent. The gold nanoparticles of DryCure Au-J were dispersed in a water–glycerol mixture. The lower surface tension of ethylene glycol may have caused a disconnection between the ink on the top of the solder mask resist and the ink in the via opening. The largest proportion of the ink flowed downwards into the via opening without adhesion to the wall of the via opening. As a result, the wall coverage was insufficient, resulting in defects and high resistances. A ramp of residual solder mask can bridge the difference in height and aid the formation of a conductive track. A higher number of layers cannot completely compensate the poor wall coverage, but increases the risk of merging of excessive ink. The higher surface tension of the water–glycerol mixture may prevent disconnection between the ink on top of the solder mask and ink in the via opening. The influence of surface tension and wettability of solder mask on wall coverage will be investigated in future work. 

Defect-free tracks and connections were achieved by gold ink printing of a minimum of two layers. Printing of only one layer resulted in very low track thicknesses insufficient for reliable track formation. The variation between circular and rectangular openings as well as the influence of additional numbers of layers was marginal. One assumption for higher standard deviation in resistance values for five printed layers was the increased risk of micro crack formation with increasing metal thickness due to thermo-mechanical stresses during sintering. Further analysis, such as thermal shock or humidity reliability testing, is needed to determine the influencing factors on resistance values and the possible cause of failures.

## 5. Conclusions

This publication presented the successful embedding of ultrathin silicon chips on polyimide foil in mechanically flexible solder mask resist and their electrical contacting by inkjet printing. The solder mask resist, manufactured for screen printing, was diluted and spray coated on epoxy bonded ultrathin chips on foil. The solder mask resist was photolithographically structured by UV-LED direct light exposure and developed to form openings above the chip contact pads. Opening sizes of 90 µm and 130 µm, as well as circular and rectangular opening layouts, were applied. For electrical contacting of chip contact pads, tracks were inkjet printed using commercial inks with silver nanoparticles as well as gold nanoparticles. Layer counts of one layer, two layers, three layers as well as five layers were printed with both inks.

The comparison of resistance values showed generally lower resistances and lower standard deviations for the gold ink DryCure Au-J. Similar to the silver ink Sicrys I30EG-1, the 90 µm openings showed better results than 130 µm openings probably due to the residual material in the openings. In general, printing of more than one layer was necessary to achieve reliable track formation. Printing of five layers of gold ink mostly increased the standard deviation of resistance compared to three printed layers. Regarding also the economic aspects, printing of two gold ink layers is the most efficient way. The presented processes are especially suited for fast development and rapid prototyping of SiF applications due to the utilization of conformal coating, direct light imaging and inkjet printing. Photolithographic layouts, as well as inkjet printed track layouts, can be altered quickly due to direct light imaging as a mask-less digital process. Spray-coated areas, direct light-exposed areas as well as inkjet-printed tracks originating from a digital layout defined in a software without additional tools or masks for layout shaping. Thereby, SiF can be manufactured in various configurations for comparably low costs and short time compared to existing technologies.

In the future, ultrathin glass as the mechanically flexible substrate could be investigated as an alternative substrate material, especially for sensor applications in high temperature environments [31]. The proposed fabrication process was also interesting for embedding and electrical contacting of ultrathin electronics grown on mechanically flexible substrates such as thin metals [32], thinned semiconductors [33] or ultrathin ceramics [34], achieving SiF with very low overall thicknesses suitable for various environments and sensor applications. 

## Figures and Tables

**Table 1:** Conformal coating parameters.

Parameter	Value
Mixing ratio solder mask resist: PGMEA	1:1.25 wt%
Valve distance over substrate	20 mm
Track distance	15 mm
Cartridge application pressure	1.5 bar
Atomizer pressure	0.5 bar
Valve movement velocity	450 mm/s
Substrate table temperature	50 °C
Dwell time after coating on substrate table	5 min
Hotplate temperature	80 °C
Dwell time on hotplate	10 min

**Table 2:** Exposure and development parameters.

Parameter	Value
LED current value (0–255)	120 (no unit)
Velocity (100–8000)	100 (no unit)
Exposure dose	1130 mJ/cm^2^
Dwell time	5 min
Developer	K_2_CO_3_ (0.8% aqueous solution)
Development time	30 s
Development temperature	30 °C
Thermal curing duration	60 min
Thermal curing temperature	150 °C

**Table 3:** Results of electrical characterization of transition resistances of printed silver tracks.

Transition Resistances Ink: Silver		Number of Layers			
		1 Layer	2 Layers	3 Layers	5 Layers
Circular openings	90 µm	211.3 Ω ± 121.1 Ω	189.6 Ω ± 299 Ω	47 Ω ± 36.1 Ω	51.1 Ω ± 30.4 Ω
	130 µm	19 defects: 11 MΩ ± 1.4 MΩ	2 defects: 33.4 MΩ ± 124.1 MΩ	2.2 kΩ ± 5.8 Ω	129 Ω ± 109.6 Ω
Rectangular openings	90 × 90 µm^2^	64.3 Ω ± 19.2 Ω	44.5 Ω ± 24 Ω	69.6 Ω ± 87.7 Ω	36.4 Ω ± 21.4 Ω
	130 × 130 µm^2^	14 defects: 6.2 MΩ ± 8.7 MΩ	4 defects: 1.8 MΩ ± 3.8 MΩ	399.5 kΩ ± 1.7 MΩ	28.9 kΩ ± 129.7 kΩ

**Table 4:** Results of electrical characterization of transition resistances of printed gold tracks.

Transition Resistances Ink: Gold		Number of Layers			
		1 Layer	2 Layers	3 Layers	5 Layers
Circular openings	90 µm	33.4 kΩ ± 152.7 kΩ	18.8 Ω ± 5.9 Ω	15.6 Ω ± 9.5 Ω	12.9 Ω ± 7 Ω
	130 µm	1 defect: 1.2 MΩ ± 2.3 MΩ	16.7 Ω ± 6 Ω	11.9 Ω ± 3 Ω	9.9 Ω ± 3.7 Ω
Rectangular openings	90 × 90 µm^2^	287.2 kΩ ± 591 kΩ	16.7 Ω ± 6.3 Ω	14 Ω ± 8.9 Ω	14 Ω ± 11.2 Ω
	130 × 130 µm^2^	3 defects: 5.8 MΩ ± 8.8 MΩ	14.9 Ω ± 3.9 Ω	9.6 Ω ± 2.6 Ω	9.6 Ω ± 9.6 Ω
